# Immediate Root Filling

**Published:** 1903-11-15

**Authors:** O. E. Inglis


### IMMEDIATE ROOT FILLING.

BY O. E. INGLIS, D.D.S.

### EXPERIMENTS IN IMMEDIATE ROOT FILLINGS.

In the college clinic I gave instructions to students to fill roots permanently and immediately after extirpation of the pulp by cocaine-pressure anesthesia and after the use of arsenic, which method was then most commonly in use. There was almost absolute success when directions were followed, and such teeth as gave discomfort were found to have done so as the result of imperfect manipulation of the root filling. In a few cases the roots were found only partly filled and proper filling produced the desired good results. For the most part in these cases cones rolled from temporary stopping were packed in sections into the canal which was moistened with eucalyptol.

The same results have been found in my own patients. In this class of cases I have used immediate filling for many years except where much apical irritation has resulted from broaching. I now, however, do not regard this as even a bar if forma-percha be used, of which I shall speak further on. In cases of aborted apical abscess, the teeth were left open after venting and root-canal sterilization until such time as subsidence of the apical inflammation occurred.

By this time the canals were again infected and sterilization had to be resorted to. In some of these cases the tentative method was used and in others immediate filling was ordered employed as for uncomplicated gangrenous pulp.

METHOD OF STERILIZING ROOT-CANALS.

In the cases of uncomplicated gangrenous pulp, the teeth were sometimes treated with the rubber dam in place and sometimes without it, as for example in cases in which the dam could not be successfully employed to exclude saliva. In the latter cases the mouth was sterilized with a strong potassium permanganate solution. Next the teeth were opened with burs until access to canals was obtained. The cavity was flushed out, the patient requested to open the mouth and the excess of moisture removed from the bulb of the pulp cavity. Previously a little sodium dioxide in powdered form was placed on a glass mixing-slab and a drop of water was also placed on the slab near it. A fine Donaldson cleanser was now drawn through the water then through the sodium dioxide and the adherent mass carried into the root-canal and gently worked in for a short distance. The sodium dioxide reacted with the water and produced sodium hydrate and hydrogen dioxide which liberated nascent oxygen. When a portion of the canal was sterilized, a Moffet syringe was held in the left hand and while gently broaching with the right hand a stream of water was passed in. The combined action freed the canal of remnants of pulp, etc., as far as the broach was inserted. The mouth was now again evacuated, the cavity again dried and more sodium dioxide introduced and carried further in. If during the course of sterilization saliva began to enter the cavity, it was removed by a douching from the syringe and work again resumed. No attempt was made to prevent a deep action of the sodium dioxide, in fact it was invited for the fine roots, yet not carried too far into the open foramen. If possible, the foramen was explored with the broach. This work was contiued for possibly ten minutes with a fairly open root; the other cases required a longer time, and, if necessary, drills and sulphuric acid were used to enlarge the root-canals.

The mechanical work and sterilization completed, the mouth was napkined to exclude moisture; the roots were dried with cotton twist on tempered Swiss broaches, thoroughly dried with hot air and filled permanently, or if desired, tentatively dressed. The permanent fillings experimented with were chloro-percha on cotton twists, gutta-percha cones in sections, the canal being moistened with eucalyptol, temporary stopping cones used in the same manner and in certain doubtful cases forma-percha on cotton.

In careful hands, good results were obtained, and in my own practice I have enjoyed the satisfaction of having no particular trouble with the method, any resulting irritation usually passing away promptly and only occasionally iodine counter-irritation being required.

### FORMA-PERCHA.

For about a year I have had pleasure in the use of forma-percha in the canals of molars. This material was introduced by Dr. John C. Blair, of Louisville, Ky., and he now states that it is composed of red base-plate gutta-percha, oil of eucalyptus, oil of cassia and paraform. As it comes for use it is in a stable, thick solution or semi-solid of friable nature. It may be taken upon a spatula and gently warmed over a flame into a thick creamy mass, which does not quickly return to the friable state. In this may be dipped a twist of cotton rolled on a fine Swiss broach, or other suitable cotton carrier, which is at once carried into the sterilized and dried canal. On the Swiss broach it is carried in with a twist to the right which screws the cotton into the canal. When placed, the broach is turned twice to the left which disengages the broach from the cotton fibers. It is withdrawn a sixteenth of an inch and the cotton crimped upon itself to pack it firmly into the root. This is repeated until the cotton is all firmly packed in. To do this successfully requires that the pointed end of the broach be cut off with scissors before rolling the cotton upon it. After this operation a cone of temporary stopping is placed into the surplus of forma-percha remaining at the mouth of the canal. Any further surplus of forma-percha is now to be removed from the bulb of the pulp chamber by wiping out with cotton; then oxychloride of zinc, stiffly mixed, is carried in on a ball burnisher or other packer, is distributed with it and then further compressed with a pellet of bibulous paper or cotton.

As a rule I have used a temporary filling of pink baseplate gutta-percha as a test filling. I have tested the removability of this root filling and find it easy of accomplishment, so that in doubtful cases it may be regarded as a therapeutic dressing, or as a permanent filling according to subsequent indications.

Human skill is not equal to the task of successfully opening all fine roots to the apical foramen so that some such removable filling or tentative dressing must be employed in such cases. In the vast majority of even such canals, however, it will not require removal. It is not miscible with water and is therefore impervious to moisture; containing formaldehyde and essential oils, it is therapeutically antiseptic for a time at least, and therefore displaces temporary antiseptics, and it is mechanically antiseptic. While it is not now possible to give it a twenty-year reputation, it may be said that Blair has given cotton and forma-percha an eight-year one. I feel that it deserves recommendation for the troublesome class of cases I have described.

### METHODS COMPARED.

The comparison of the tentative and immediate methods in the same case deserves some notice. I will mention a few typical cases. In a lower bicuspid with gangrenous pulp I opened under rubber dam, sterilized and dressed with cotton and an antiseptic. After two days the patient reported a slight soreness. I dressed again; still there was some tenderness and so on until I became disgusted. I then sterilized and filled the root permanently and applied iodine to the gum. After a slight soreness the tooth became comfortable and so remains.

An upper central incisor with a history of apical pericementitis was sterilized with sodium dioxide and dressed with cotton and an antiseptic. In a few days it was opened and a free flow of pus followed the removal of the cotton. I again sterilized for about fifteen minutes with sodium dioxide, forcing it well into the apical space. After drying thoroughly, I moistened the canal with eucalyptus and filled with sections of a cone of temporary stopping. Iodine was then applied to the gum. This case was absolutely comfortable from date of filling and was under observation for two months after filling. It did not form a fistula nor show any sign of pericemental irritation. In that class of cases in which effusions continually escape from the apical tissue into the canal as is shown by the repeated moistening of the cotton swab, there is no doubt that from that point immediate work is of as great value as a tentative method. Deliquesced zinc chloride in very small quantity on the end of the cotton swab will, as a rule, promptly check the effusion, when the canal may be dried and filled. I know that this use of zinc chloride has been critised, but I beg to report my own good results notwithstanding.

Immediate filling in cases of certain forms of apical abscess of semi-chronic or sub-acute nature seems good in my experience, but I am not prepared to advise its use in the violently acute cases. In cases of chronic abscess with fistula, it is good practice and at times seems to be the only method of effecting a cure, though to be sure there are cases of septic cementum that are beyond a cure by this method. Aristo-paraffin, aristol and wax, and paraform and wax are other forms of removable root fillings and in the larger roots any of the forms of gutta-percha are quite readily removable by means of drills; cotton and chloro-percha is the most difficult of them to remove.

The time saved by immediate root filling is enormous, and in full practice it is a great comfort not to be compelled to sandwich patients into one's engaged hours for the purpose of treating teeth. The patients will come out of the proper time in spite of every precaution and the earning capacity of time is markedly lessened without a compensating remuneration from the treating. While no one would willfully neglect the requirements of any case or treat immmediately when circumstances indicate another course, this is a point of no mean importance. As stated by one of my confreres across the club sandwich, "One does a lot of little things in treating which tax his ingenuity and take up his time and for which he does not get paid." He is a man who thinks well of himself and has a fine practice among well-to-do and discriminating patients so that I think my ground is fairly well taken.—*Items of Interest.*

